# General practice-based undergraduate pharmacy longitudinal clerkship: a theoretically underpinned qualitative evaluation

**DOI:** 10.1007/s11096-022-01429-0

**Published:** 2022-07-26

**Authors:** C. Innes, S. Cunningham, B. Addison, Y. Wedekind, E. Watson, I. Rudd, A. Power, L. Karim, G. F. Rushworth

**Affiliations:** 1 School of Pharmacy and Life Sciences, Robert Gordon University, Aberdeen, AB10 7GJ UK; 2 Highland Pharmacy Education & Research Centre, NHS Highland, Inverness, IV2 3JH UK; 3 School of Medicine, University of Dundee, Nethergate, Dundee, DD1 4HN Scotland UK; 4 NHS Highland, Old Perth Road, Inverness, IV2 3JH UK; 5 Central Quay, NHS Education for Scotland, 89 Hydepark Street, Glasgow, G3 8BW UK

**Keywords:** Clinical clerkship, Experiential learning, General practice, Pharmacy

## Abstract

**Background:**

A Pharmacy Longitudinal Clerkship (PLC) was designed to develop student pharmacists’ (SPs) competence in a general practice setting.

**Aim:**

The aim was to carry out a theoretically underpinned qualitative evaluation of stakeholder perceptions of influences of behavioural determinants on SP development for clinical practice in general practice.

**Method:**

General practice-based PLCs were delivered in 2019/20 and 2020/21 for two cohorts of SPs in NHS Highland, Scotland. Qualitative semi-structured interviews were used to explore stakeholder perceptions of influences of behavioural determinants on SP development. Informed written consent was obtained. An interview schedule was developed and piloted using the Theoretical Domains Framework (TDF). Interviews were recorded, transcribed verbatim and analysed using thematic methodology. Ethics approval was granted.

**Results:**

Seven SPs and five general practitioner (GP) tutors were interviewed. Key themes were identified mapped to TDF domains and included: knowledge—utilisation and practical application of knowledge; skills—triangulation of skills under clinical supervision; beliefs about capabilities—confidence building with clinical and patient contact; professional role and identity—elucidation of professional roles within general practice.

**Conclusion:**

This evaluation shows benefits of embedding SPs within clinical teams and immersing them in a clinical environment over a prolonged period in a general practice Pharmacy Longitudinal Clerkship. It is expected this will translate into a more confident transition to postgraduate professional clinical practice. Funding should be sought to test alternative PLC arrangements including: multiple full-time longitudinal placement blocks; or ultimately a year-long longitudinal clerkship programme with an IPE element.

## Impact Statements

- Pharmacy Longitudinal Clerkships in general practice, which include supervised clinical practice, increase student pharmacist confidence in managing real-life clinical presentations.
- Prolonged supervised practical clinical placement experience allows utilisation of existing clinical knowledge as well as development and application of clinical skills sets.
- Embedding student pharmacists in multidisciplinary teams has benefits across the professional spectrum in terms of triangulation of professional roles and boundaries.

## Introduction

The role of qualified pharmacists is continually being developed across the world. In a recent global survey, 28/48 (58%) countries reported that advanced practice frameworks were currently in use or being developed within their country [1]. Within the UK, post-registration curricula from the Royal Pharmaceutical Society (RPS) have defined the standards required of patient-facing pharmacists working at Foundation [2], Advanced [3] and Consultant [4] levels of practice.

The General Pharmaceutical Council (GPhC—UK Pharmacist Regulator) recently published Initial Education and Training (IET) Standards which, from summer 2026 onwards, permits pharmacists to have prescribing rights at point of registration [5]. If patients and the public are to be assured of the quality of a prescribing pharmacist clinician workforce, then the delivery of pharmacist IET needs to be significantly augmented and adapted to optimise the training environment. Exposure of student pharmacists (SP) to clinical learning environments is anticipated to increase the practical knowledge, skills and behaviours of the postgraduate and post-registration workforce such that they will be able to competently and autonomously manage patients, their diseases and medicines safely from an earlier stage in their professional career and be in a position to begin to develop advanced roles [6] sooner.

Medical Longitudinal Integrated Clerkship (LIC) models have been used across the world in countries such as Australia, Canada, USA and South Africa [7, 8]. LICs have generated an evidence base which supports transformational practice and workforce outcomes within these countries. LICs afford medical students a longer period of continuous time in the same practice and as a result medical students reported greater understanding and appreciation of general practice careers as a benefit [9, 10].

Definitions and examples of longitudinal clerkship-type models within pharmacy from across the world are limited. Within Ireland, Kerr et al. reported on an evaluation of a longitudinal placement for 2nd year students within a 5 year pharmacy degree and concluded that it promotes learning through curricular integration and interdisciplinary collaboration [11]. These placements related to SPs spending one half-day per week spread over a 13 week semester and were based in the community pharmacy setting.

In 2017, a Pharmacy Longitudinal Clerkship (PLC) programme was announced which offered opportunities for EL and interprofessional learning as well as the development of clinical and consultation skills in managing ‘real-life’ practice-based clinical scenarios that meet the aspirations for service delivery models of the future [12]. A 10-week PLC pilot, involving two fourth-year SPs from the Robert Gordon University, was completed from November 2018 until January 2019 [13]. SPs were in practice for 3 days each week. The pilot reported increased student confidence in clinical practice, increased enthusiasm for a career in pharmacy as well as General Practitioner positivity for the PLC compared with more traditional limited-duration placements. Building on the learning from the pilot, two further PLC cohorts were evaluated in academic years 2019/20 and 2020/21—the results of these two further cohorts are published in this paper.

### Aim

The aim was to carry out a theoretically underpinned qualitative evaluation of stakeholder perceptions of influences of behavioural determinants on SP development for clinical practice in general practice.

### Ethics approval

Ethics approval was sought and granted by the Robert Gordon University School of Pharmacy and Life Sciences ethics review committee (Ethics Approval Number: S150; 20th November 2018).

## Method

This research was undertaken according to an interpretivism philosophy using a qualitative research methodology. Conducting individual semi-structured qualitative interviews with PLC participants was considered the most appropriate method to facilitate in-depth rich data capture and analysis.

### Setting

The PLC was based in five GP training practices in the NHS Highland area of Scotland. These GP practices covered a range of rurality based upon the Scottish Government eightfold Urban Rural Classification [14]. Two practices were in the “other urban area”; one in the “accessible small town”; one in a “remote small town”; and one in a “remote rural” location. Practice list sizes ranged from around 5000 to 15,000 patients.

### Sampling and recruitment

PLC was run as a research programme such that Student and GP recruitment onto the educational programme was intrinsically linked with research recruitment. Student recruitment was limited to five students each year for two years based on available funding. Applications were open to the whole final-year MPharm group. Applications were shortlisted by panel prior to interview.

GP practices were recruited using a purposive sampling method where all GP training practices within NHS Highland who had experience of hosting University of Dundee LIC students were invited to participate.

All GP clinical tutors and all SP were invited via email to participate in the qualitative interviews. There were no exclusions.

### Data collection

The interview schedule for the semi-structured interview was developed from several sources: literature review; PLC pilot topic guide [13] and The Theoretical Domains Framework (TDF). The TDF summarises key elements of 33 theories and proposes that determinants of behaviour cluster into 14 domains [15]. Those domains most relevant (e.g. knowledge, beliefs about capabilities and consequences, motivation and goals, environmental context and resources) were used to guide construction of interview core questions as they allowed systematic consideration of behavioural determinants for SP development of competencies for clinical practice. The data collection process was linked directly to the views and experiences of stakeholders and their involvement in the PLC. Credibility of the interview schedule was enhanced through review of the draft by key expert researchers and practitioners.

Each stakeholder was provided with study information sheet prior to obtaining informed consent. The researchers (CI and TJ) trained in carrying out individual semi-structured interviews conducted either face-to-face in the workplace or telephone interviews, depending on what was convenient for the participants.

### Data analysis

Qualitative interviews were audio-recorded, transcribed naturalistically verbatim, and checked for accuracy by the research team. The data generated were analysed thematically [16] using an interpretivist approach. Data was uploaded to NVIVO to facilitate coding. The initial coding framework developed based on the main sections of the interview schedule and using the TDF domains as a thematic guide. This was then applied to one interview by different researchers. This was then modified and refined and applied to subsequent interviews independently by two experienced qualitative researchers (CI and LK). The processes and stages of analysis were overseen at all stages by a Professor of Pharmacy (SC) who mediated on any disagreements that could not be resolved through discussion by the analysis team.

Illustrative quotes were selected through team discussion. The quotes and themes were used to produce a textural description of the theme and a structural description of the context or setting that influenced participants’ experience. These were then used to create a composite statement of the “essence” of the evaluation themes.

Saturation of themes was explored by considering the adequacy of the number of interviews in each group and was considered to be reached when no new themes emerged [17]. Credibility was enhanced through pilot testing of the interview schedule, audio recording and transcription checks, independent coding, and use of verbatim quotations. Transferability was assured through detailed reporting of the research process and inclusion of characteristics of the interviewees and their settings. Dependability arose within the process of data analysis through repeated listening and review of transcripts. Lastly confirmability was enhanced through regular meeting and discussion of the research processes with the whole research team and through inclusion of verbatim illustrative quotes from participants to support the thematic narrative.

## Results

Five SPs were recruited to the 2019/20 cohort; of these, three SPs completed the ten-week block and two SPs partially completed the placement, leaving after 6 weeks. Five SPs were recruited to the 2020/21 cohort; of these, two SPs were unable to start due to international travel restrictions imposed during COVID. The duration of the 2020/21 PLC also had to be shortened due to COVID quarantine requirements prior to attendance on placement and was expected to run for eight weeks in total. Of the three SPs in 2020/21 undertaking PLC: one SP completed the eight week PLC, one SP completed six weeks, and the other SP completed five weeks. PLC had to be stopped early for the last two students due to Scottish Government legislation limiting travel during the COVID pandemic. Neither student willingly dropped out. All SPs spent three days per week in practice and the other two days on coursework while on the PLC.

Individual qualitative semi-structured interviews were conducted with seven SPs (19/20 cohort *n* = 4; 20/21 cohort *n* = 3) and five GP tutors. One GP tutor was unable to be contemporaneously interviewed due to COVID workload. Two of the five GP tutors were involved with both cohorts and so were interviewed once. 2019–20 interviews: one GP was interviewed in person, three GPs by phone, four SPs in person. All 2020–21 interviews were completed by phone: one GP and three SPs. GP interview mean 42 min (range 35–50 min); SP interview mean 57 min (range 43–78 min).

All GP tutors had prior experience with undergraduate medical students, and one had co-authored the NHS Education for Scotland Advanced General Practice Clinical Pharmacist Framework [19]. However, only one had experience of supervising SPs, but not as lead tutor. Some GP tutors (*n* = 3) had prior experience of being tutors for medical LIC students.

In general, participation in the PLC was a positive experience for SPs, including those that withdrew before the end of the placement. This was reflected throughout the interviews. The main themes identified related to: utilisation and practical application of knowledge; triangulation of skills under clinical supervision; confidence building with clinical and patient contact; elucidation of professional roles within GP.

Key themes emerging from the focus group, linked to the TDF, are given in Table 1.

**Table 1:** Key themes emerging from focus groups

TDF domains	Overarching theme	Description	Supporting quotes
Knowledge	Utilisation and practical application of knowledge	Most of the SPs identified that participating in the PLC had helped them apply their knowledge, realising that applying it with patients was different from theoretical or simulated cases in the university environment	"I wasn't sure about the application of the knowledge in practice and, I think that extended period of time, where I was exposed to all of that—putting names to illnesses and putting faces to illnesses—just consolidated all that, […] work from university, for me” [SP1 2019–20]
		The most frequently identified gap in knowledge cited by students was “linking” different elements of knowledge together to inform clinical reasoning and decision making and informing diagnosis, management and prescribing	“It was about bringing that knowledge and understanding of a single disease state and trying to group it so that you know what to question to kind of rule out the most serious illnesses” [SP2 2019–20]
		Other identified gaps included depth of knowledge and insight into unknown unknowns	“I realised early on that there was definitely gaps in my knowledge of things I'd already learned, I just didn't know” [SP3 2020–21]
		One GP tutor was very impressed with the pharmacy students’ knowledge and professional manner throughout her placement. However, the opinion on pharmacy students’ knowledge did vary across the GP tutors	“It was fantastic the knowledge they did bring actually … the pharmacy knowledge. I mean, that was actually really helpful, […] to bring their expertise, and I think the patients really appreciated that as well.” [GP4 2019–20]
		SPs also reported how supportive the GP Practice learning environment was:	“For anything that I didn't know, or asked a question about, I was never made, I was always made to feel very included and, yeah, I was never made to feel bad for not knowing “ [SP2 2020–21]
Skills	Triangulation of skills under clinical supervision	Students also discussed that the PLC helped with application of skills as well as knowledge and recognised that practise of skills within a practice environment is required	“[I]didn't know the scope of… why you were taking them [basic observations] and how they affected, and how they could be abnormal and what that may indicate, and I think, definitely, PLC gave me great understanding of that” [SP3 2020–21]
		All students reported participation in PLC had improved their communication and consultation skills	“I feel like mine [communication skills] have come on so much from having my time there, both kind of professionally and with patients” [SP2 2020–21] “[consultation skills] was something I kind of struggled with initially, to just get the nice balance of professionalism but also keeping it natural as well. But they were all definitely enhanced while I got to spend time in [the GP practice]” [SP3 2020–21]
		One tutor was very positive about their student’s consultation skills with patients and their interactions within the multidisciplinary team	“[they were] absolutely fantastic at making patients feel at ease. [They were] very human, very real and… just a very caring healthcare professional. Extremely approachable; patients all really liked [them], and [they were] really professional and appropriate with everyone, and very eager and keen to learn” [GP1 2020–21]
		Some students also noted the rapid development of practical clinical assessment skills and, for some, the confidence that this gave them:	“…in practice, it happens so quickly, one morning you feel like you really can't do something and by the end of the afternoon you're sort of, oh, that's fine.” [SP2 2019–20]
		One student commented that they benefited from:	“… seeing patients and sort of thinking about them more from like the holistic point of view, rather than just what’s on their prescription” [SP3 2019–20]
		However, one GP expressed that the students could have had better preparation prior to starting PLC – particularly with regards to history taking and succinctly presenting cases:	“Our second student […] probably didn't come with that same experience … and therefore … the most challenging aspect for [them] was actually gaining some confidence and some skills in that patient facing consultation [….] I saw progress on that during the course of [their] time here.” [GP3 2019–20]
Beliefs about Capabilities	Confidence building with clinical and patient contact	The majority of students reported an increase in confidence having participated in the PLC, with one student commenting:	“If it was on a zero to hundred scale, I think I’m like a hundred times more confident than I was going into the clerkship” [SP1 2019–20]
		Students acknowledged that applying and consolidating knowledge and skills during the placement led to increased confidence:	“I think it is about having that confidence kind of in yourself which is quite difficult to start with, to be, you know, there was a few times that folk asked me questions and I was like, oh, I don't know, and then they would tell me the answer and I'd be like, oh, I did know that.” [SP2 2020–21] “There was quite a few red flags that I might not have picked up on if someone had said to me, or related to a problem, but now I think I'd be a lot more comfortable to do that” [SP2 2020–21]
		One student felt that, in addition to her professional confidence, her self-confidence had also increased commenting:	“I think you come out stronger at the end in so many different ways. In terms of your […] clinical skills […] and then like aspects of your own personality as well” [SP3 2020–21]
Professional Roles & Identity	Elucidation of professional roles within GP	Most GP tutors saw their role as a supervisory, overseeing the placement and offering support when needed:	“It was just making sure that [they were] okay and adapting the programme to what [they] felt [they] needed at different stages” [GP2 2019–20] “…trying to give them a good glimpse of general practice, and to enthuse them, in a way, and to get them to understand the complexities of a primary care team” [GP3 2019–20]
		The importance of feedback and encouragement from their tutor in addition to exposure to tasks was identified, which led to increased confidence of the students with one commenting:	“I think just the exposure to doing it and, from feedback off my tutor as well, saying, like, it's okay, you are doing the right thing” [SP4 2019–20]
		One GP tutor also suspected that this opportunity gave their student a different understanding of a pharmacist’s role in practice:	“It gave [them] insight into what you could do as a pharmacist, and certainly [what they] expressed that at the end, is that [they would] never would of thought that [they] would've ended up doing the things that [they] did, and that seeing pharmacists in advanced roles suddenly made [them] think about [their] career in a different way” [GP3 2019–20]
Environmental Context and Resources	Influence of environmental context and resources	For the majority of SPs the PLC was perceived as a positive learning experience:	“It was just such a supportive environment and I just really, really, I think the way they let me decide when I was ready to do something, but at the same time, gave me an idea of when I should be able to do it. It was just a really nice balance, and I think that actually made me flourish more” [SP3 2020–21]
		However, an issue was noted at one site where there was a miss-match of expectations around the knowledge and skill base of SPs:	“Perhaps maybe it was … an issue to do with their [GPs] confidence in what a pharmacy student knew. I think it was a sort of [a lack of] awareness [from the GP].” [SP3 2019–20]
		This was echoed by the student’s GP tutor who commented:	“It was difficult to know what [their] knowledge base was meant to be” and “maybe a bit more information about what their training involves, in comparison to medical students… maybe pointing out the differences between the two…” [GP2 2019–20]
		One student also reported developing an increased awareness of the importance of lifelong learning commenting:	“It wasn't until I actually started PLC I realised that … seeing other healthcare professionals, people that were already graduated and were working years, and their attitude towards it was that you're learning, and you can always learn something new” [SP3 2020–21]
		It was noted by some of the student pharmacists that interprofessional learning with non-pharmacist healthcare professionals added depth to their learning:	“I think you get definitely a lot, a better range of things to think about and it definitely broadens your mind working with people that are [in] healthcare but different professions” [SP3 2020–21] “I think the main benefit was that the teaching was different from [that of] a pharmacist, I think. Whenever I've been with a pharmacist it's a lot of like: a question–answer, question–answer, because you know the medication or don't, and with the doctors, it's a bit more holistic” [SP2 2019–20]
		It was identified the importance of supporting the SPs, and encouraging them to work as part of the multidisciplinary team. All GP tutors reported it was important for the student to experience as many of the different health disciplines as possible, to give them the best experience of general practice	“We tried to really give [them] a chance to experience everything. [They were] offered, a session with midwives, you know, with physios, occupational therapists, the pharmacy team, the social work, district nurses, nurses here, practice nurses, healthcare assistants, doctors, home visits, hospital management, discharge management, terminal care, communication skills, you name it. The front desk, reception, answering phones, gathering information […] we made sure that, as much as possible, [they] get exposure to every single one of these scenarios” [GP1 2020–21]

## Discussion

### Statement of key findings

This work provides data on the significant and unique opportunities for SPs of a GP longitudinal clerkship EL placement. Clear benefits were seen by both SPs and GP tutors of embedding SPs in clinical practice for prolonged placement. Key findings related to the five main themes.

*Utilisation and practical application of knowledge*: there were clear benefits reported by students of applying knowledge in clinical practice in real patients rather than in simulated cases. Some specific benefits related to the necessity to link data and complexity in real life patients and students gaining an insight to the limitations of their own knowledge in a practical context and well as having the opportunity to explore their “unknown unknowns” (things they did not know that they did not know) with a GP tutor.

*Triangulation of skills under clinical supervision*: again, students also reported benefits of practicing clinical skills with patients and in particular consultations skills. Some GPs commented on their perceptions of how the lack of clinical consultation exposure prior to this placement made the PLC challenging for their SPs.

*Confidence building with clinical and patient contact*: there was an overwhelmingly positive response from students in terms of the benefit of prolonged clinical exposure on their clinical confidence. This confidence seemed to extend beyond simple confidence in their clinical and consultations skills but was also felt to have increased their personal self-confidence too.

*Elucidation of professional roles within General Practice:* being embedded in a GP clinical team and having input from a GP as well as witnessing advanced pharmacists in practice had a profound effect on some whereby this experience opened their minds to the challenges and complexities of general practice.

*Influence of environmental context and resources:* there were however some learning points to take away around setting expectations of SPs knowledge and what the training involves and could deliver within the timescales. However, overall the SPs seemed to relish the opportunity to learn in practice within the multidisciplinary team and benefited from this type of exposure.

PLC has demonstrated the benefits of teaching and application of clinical and communication skills within the practice setting, with ease of access to patients and the wider healthcare team, rather than in the setting of a university campus.

### Strengths and limitations

A key strength of this study is that it provides valuable information on SP and GP tutor views and experiences of a PLC. Such information is lacking in the published literature. It is important to consider the effect of reflexivity in the qualitative research process and consider the impact personal experiences may have on the outcomes of this research. To minimise this, the trustworthiness of the qualitative research process has been strengthened by reporting the study in line with COREQ guidelines [18].

Limitations of this work include the small number of participants and the effect of COVID regulations on participants’ ability to undertake the full placement. However, those adversely affected by COVID regulations still reported a positive learning experience. There exists the potential for bias in the collection of the data due as all SPs and GPs would know the researcher conducting the interviews. While both SPs that opted to stop the PLC early were invited to interview, only one was able to participate. Some aspects may not be directly transferable to other settings or countries.

### Interpretation

#### Utilisation and practical application of knowledge & Triangulation of skills under clinical supervision & Confidence building with clinical and patient contact

Interpretation of these first three themes has been amalgamated as there is thought to be considerable overlap in the rational for the findings.

Overall, this PLC is entirely compatible with the recently published GPhC IET Standards where there is an aspiration that pharmacists will play a much greater role in providing clinical care to patients, as clinicians [5].

Key enablers to the success of the PLC include effective preceptorship and prolonged exposure to patients and multidisciplinary teams. The positive influence of effective preceptorship on SP communication skills has been reported in the USA [19]. The effect of immersive and an ongoing continuum of preceptorship, from the same, high-quality medical preceptor, has not been evaluated elsewhere. The benefit to the development of clinical knowledge, clinical skills and patient communication skills can all be influenced by high-quality clinical preceptorship. There is also clear evidence in this research of increased SP confidence from experiential consolidation of knowledge and expansion of clinical skills. This type of increase in confidence in communication skills from placement activity has been seen in other healthcare professional groups, including undergraduate speech pathology students in Australia [20]. To create credible and useful clinicians from our pharmacy degrees, similar EL opportunities are essential which host students for substantial periods of time within clinical environments.

#### Elucidation of professional roles within general practice

Improving self-confidence in communication skills through IPE in medical, nursing and pharmacy students in the USA as part of a communication skills development course [21]. Further work has been completed within the clinical placement setting within Scotland across multiple sectors of practice [22] as well as on a one-week pilot within a secondary care setting between medical and pharmacy students [23]. A key opportunity arising from the PLC is the potential to explore the possibility of expanding IPE within experiential settings to complement campus-based education activities.

#### Influence of environmental context and resources

The suitability and acceptability of SP placements in general practice has been reported in England [24]. However, the longitudinal element of this placement was unique and the compound effects of such immersion in general practice should not be understated. Also, the context of placing the SPs with GP, rather than pharmacist mentors, should also be considered unique. While there is a definitive need for the pharmacy profession to look to reduce its reliance on GP-time, this research has also reported on the additional insight gained by GPs of a separate healthcare professional group. The effect of that exposure is beyond the scope of this research to explore. There does however remain a need for pharmacy to continue to develop high-quality pharmacist clinical supervisors such that they could take over clinical supervision responsibilities wherever possible.

### Further research

Consideration has been given to the future of the PLC by the Scottish Government. An adapted “longitudinal placement” model will now become part of the EL offered within multiple pharmacy sectors in any remote and rural area within Scotland. To fit with timetabling constraints these “longitudinal placements” will occur at the same site over multiple separate full-time EL placement weeks, rather than as one continuous part-time clerkship placement block. Further research is required to determine the effect of this change in structure.

Given the success of PLC in terms of meeting the objectives of developing a SP clinician product, further pilot schemes should be considered which would see SPs given similar EL placement opportunities to other student healthcare professionals. Year-long clerkships at undergraduate level should be piloted to explore the benefits of entirely immersive EL clerkships for SPs. Given the pre-existing medical clerkships and the positivity around interprofessional working outlined, consideration should be given by Scottish Government and NES as to IPE longitudinal clerkships.

Further research should focus on interprofessional education opportunities within PLC and longitudinal follow-up of SPs to determine the success of the programme.

### Conclusion

This research demonstrates the benefits of embedding SPs within clinical teams and immersing them in a clinical environment over a prolonged period of time as part of a Pharmacy Longitudinal Clerkship model within General Practice. Responses were positive in terms of SP and GP tutor experience in relation to application and triangulation of knowledge and skills under clinical supervision. It built confidence in SPs in relation to practical application of their clinical knowledge, skills and behaviours which will ease their transition to postgraduate professional clinical practice.

Immediate adoption of PLC, as proposed, will see repeated exposure of SPs during EL weeks throughout the year. However, funding should be sought to test alternative PLC arrangements including longer full-time blocks or ultimately year-long EL placements with an IPE element for SPs should be considered.
